# HSPB8 overexpression prevents disruption of blood-brain barrier after intracerebral hemorrhage in rats through Akt/GSK3β/β-catenin signaling pathway

**DOI:** 10.18632/aging.103773

**Published:** 2020-09-04

**Authors:** Ying Hou, Zhiping Hu, Xiyu Gong, Binbin Yang

**Affiliations:** 1 Department of Neurology, 2nd Xiangya Hospital, Central South University Changsha, Hunan Province, China

**Keywords:** HSPB8, intracerebroventricular injection, ICH, BBB, Akt/GSK3β/β-catenin pathway

## Abstract

Blood brain barrier (BBB) disruption is a crucial factor contributing to secondary brain injury after intracerebral hemorrhage (ICH). Heat shock protein B8 (HSPB8) has been recently reported to confer neuroprotection against against ischaemic stroke through maintaining BBB integrity. However, the role of HSPB8 in ICH is still elusive. In this study, we found that HSPB8 was upregulated by ICH and extensively expressed in neurovascular structure including endothelial cells and astrocytes. lentivirus intracerebroventricular (*i.c.v*) injection achieved a widespread and persistent HSPB8 overexpression in brain tissues. HSPB8 overexpression significantly ameliorated neurobehavioral deficits and brain edema at 24 and 72h following ICH. Moreover, HSPB8 overexpression remarkedly inhibited BBB disruption and significantly increase the level of p-Akt, p-GSKβ and intranuclear β-catenin 24h post-ICH. This effect was obviously reversed by Akt specific inhibitor, MK2206. Based on these findings, HSPB8 exerted its protective effect on BBB, at least partly, via Akt/ p-GSKβ/β-catenin pathways.

## INTRODUCTION

Spontaneous intracerebral hemorrhage(ICH), caused by the rupture of blood vessels in the brain, is the most devastating type of stroke [1, 2]. It is becoming increasingly clear that BBB disruption is one of the critical mechanisms of ICH-induced brain injury. Therefore, search for effective neuroprotective agents ameliorating BBB collapse may provide therapeutic potential for ICH.

As a member of small heat shock proteins (sHSPs) family, HSPB8 is of particular interest in the field of neurological diseases. HSPB8 has been demonstrated to interfere with detrimental processes during neurodegenerative diseases [3, 4]. Our previous investigation has reported that HSPB8 effectively conferred protection against cerebral I/R injury by improving BBB stability [5]. However, we do not yet know the possible role of HSPB8 in ICH.

Accumulating evidence has confirmed that the Wingless integrated MMTV (Wnt)/β-catenin is a major pathway important for the stabilization of brain endothelial tight junctions [6, 7]. Mounting evidence indicates that Wnt/β-catenin signaling is negatively regulated by the activity of GSK3β [8, 9]. In addition, Akt results in GSK3β inhibition through inducing GSK3β phosphorylation at Ser9 [10]. Therefore, Akt/GSK3β/β-catenin signaling may play a positive role in BBB protection. Previous studies have shown that HSPB8 activated PI3k/Akt in different disease models, including brain ischemia, chronic myocardial ischemia and hepatic carcinoma [11–13]. However, whether HSPB8 protects against BBB disruption after ICH via regulating Akt/GSK3β /β-catenin signaling pathway is largely unknown. The aim of the present study was to explore the potential role of HSPB8 in BBB maintenance in ICH rat models. We also investigated the possible mechanisms underlying HSPB8 protective effects.

## RESULTS

### Increased HSPB8 protein expression in rats after ICH

**Figure 1:**
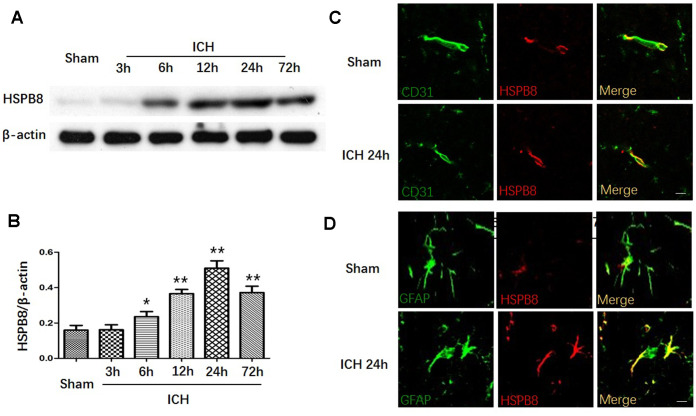
**HSPB8 expression was increased upon ICH.** (**A**) Representative western blot bands for HSPB8 expression in sham and ICH rats 3, 6, 12, 24, and 72 h following ICH. (**B**) Densitometric quantification of HSPB8 for the western blot (n=6, mean±SEM). (**C**) Representative images of immunofluorescence staining for HSPB8 and CD31 in control and ICH (**D**) Representative images of immunofluorescence staining for HSPB8 and GFAP in control and ICH. Scale bars = 50 μm (n =4, mean±SEM). *P <0.05 versus sham group; **P<0.01 versus sham group. n=number of animals per group.

We first measured the protein expression of HSPB8 in perihematomal brain tissue after ICH at different time points. In comparison to the sham group, the western blot results showed that HSPB8 protein level in the perihematomal tissues was significantly increased in the rats suffered from ICH injury from 6h post-ICH and peaked at 24h after ICH (Figure 1A, 1B). Moreover, double immunofluorescence staining showed that HSPB8 was sparsely expressed and co-localized with CD31 (endothelial), and GFAP (astrocytes)in sham group. HSPB8 immunoreactivity on neurovascular structures was increased around the hematoma 24 h after ICH (Figure 1C, 1D).

### Effect of intracerebroventricular (*i.c.v*) injection of pLV-HSPB8 on the expression of HSPB8 in brain

**Figure 2:**
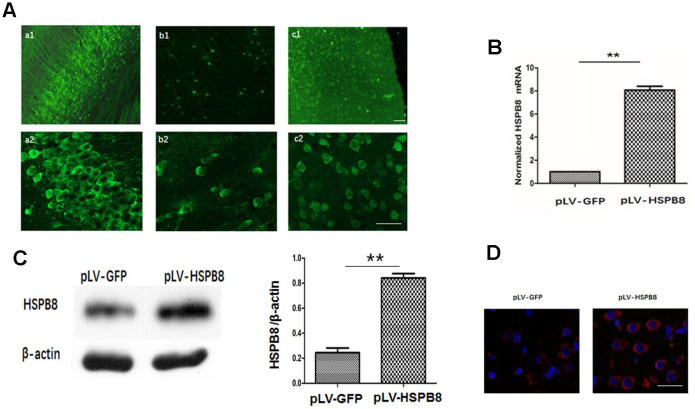
**Lentivirus *i.c.v* administration mediated HSPB8 overexpression in brain tissues.** (**A**) Representative fluorescent images labeled with anti-green fluorescent protein (GFP). a1, b1, and c1 were images at lower magnification in the hippocampus, striatum and cortex; a2, b2, and c2 were at higher magnifications. (**B**) RT-PCR result analysis (n=5, mean±SEM). (**C**) Western blotting and quantification of HSPB8 protein (n=5, mean±SEM). (**D**) Representative photomicrographs of immunostaining for HSPB8 (red) and DAPI (blue). Scale bars = 50 μm. **P<0.01 versus pLV-GFP. n=number of animals per group.

A lentiviral delivery of HSPB8 gene into cerebroventricular was performed to investigate the role of HSPB8 in ICH-induced brain injury. Two weeks following *i.c.v* intraventricular injection of pLV-HSPB8 lentivirus, widespread GFP fluorescence was observed throughout the CNS, including the cortex, hippocampus and striatum (Figure 2A). The data demonstrated that widespread delivery of the transgene was achieved using pLV vectors. To estimate level of HSPB8 in brain, we performed mRNA and western-blot. The results revealed a dramatic increase in HSPB8 gene and protein expression in the brain tissues of the experimental ICH+pLV-HSPB8 group compared with ICH+pLV-GFP group (Figure 2B, 2C). Furthermore, brain sections of animals treated with ICH+pLV-GFP showed low immunopositive HSPB8-elements, while in ICH+pLV-HSPB8 treated group, HSPB8 immunostaining was strongly expressed throughout the brain (Figure 2D). This implied that lentivirus achieved widespread infection within brain tissues with high efficiency and allowed a widespread and persistent HSPB8 overexpression.

### HSPB8 overexpression improved behavioral deficits and restrained cerebral edema at 24 and 72 h after ICH

**Figure 3:**
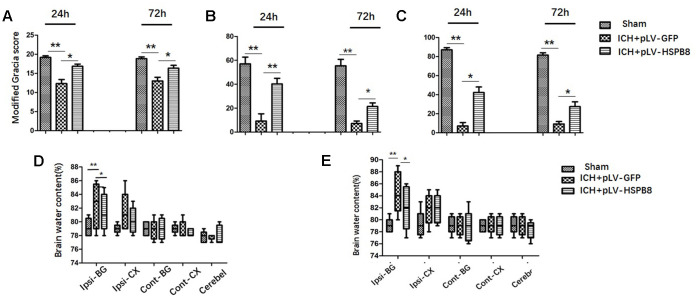
**HSPB8 overexpression significantly reduced brain injury and brain edema at 24 and 72h after ICH**. (**A**–**C**) Modified Garcia test, corner turn test, and limb placement test at 24h and 72h post-ICH in sham, ICH+ pLV-GFP or ICH+pLV-HSPB8 groups (n=6, mean±SEM). (**D**) The brain water content at 24h following surgery in sham, vehicle, and pLV-HSPB8 treatment groups. (**E**) The brain water content at 72h following surgery in different groups. Brain sections were divided into five parts: ipsilateral basal ganglia, contralateral basal ganglia, ipsilateral cortex, contralateral cortex, and cerebellum (n=6, mean±SEM). **P <0 .01 versus indicated group; *P <0.05 versus indicated group. n=number of animals per group.

Next, we investigated the effects of HSPB8 overexpression on the ICH-induced brain injury. ICH rats exhibited significant neurobehavioral deficits at modified Garcia test, performances of the forelimb placement and corner turn test at 24h after surgery when compared with sham animals. Lentivirus induced-HSPB8 overexpression markedly improved these neurobehavioral functions compared to ICH+ pLV-GFP group at 24h after ICH (Figure 3A–3C). At 24h after ICH, a significant increase in brain water content was observed in the ipsilateral cortex and basal ganglia in ICH+ pLV-GFP group compared with the sham group. There were no differences in brain water content observed between all groups in contralateral cortex, contralateral basal ganglia or in cerebellum. HSPB8 overexpression significantly reduced the water content in the ipsilateral brain basal ganglia and cortex at 24h post-ICH compared with the vehicle control (Figure 3D, 3E). The performance of ICH+ pLV-HSPB8 group on Modified Garcia, Forelimb placement, and Corner turn test was obviously improved at 72h after ICH, compared with ICH+ pLV-GFP group. Consistently, HSPB8 overexpression reduced brain water content in the ipsilateral basal ganglia significantly.

### HSPB8 overexpression rescued the blood-brain barrier impairment after ICH

**Figure 4:**
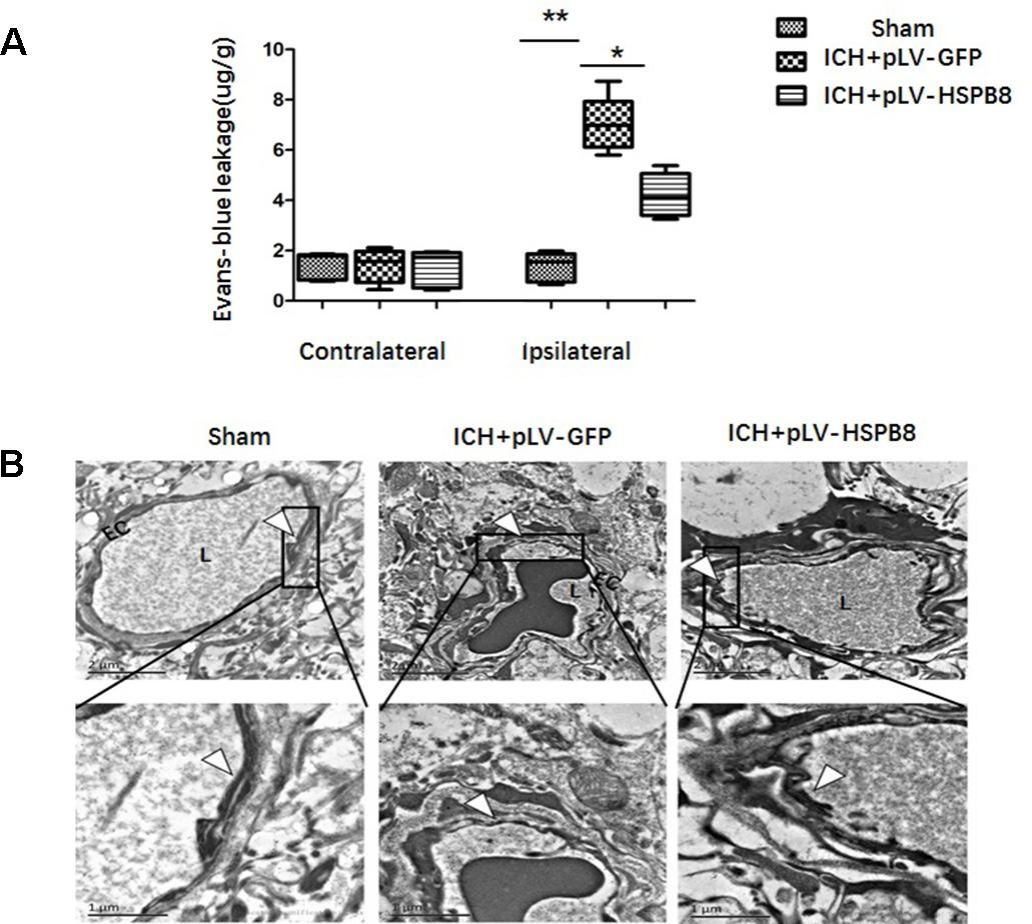
**HSPB8 overexpression inhibited ICH-induced BBB destruction.** (**A**) Quantitative analysis of Evans blue extravasation at 24h after ICH (n=5, mean±SEM). (**B**) TEM images of the morphometric changes of BBB (n=5, mean±SEM). EC: endothelial cell; L: lumina. Arrowheads show TJ. **P <0 .01 versus indicated group; *P <0.05 versus indicated group. n=number of animals per group.

BBB functionality at 24h following ICH was evaluated by Evans Blue (EB) extravasation. As shown in Figure 4A, ICH caused a significant increased dye extravasation in ipsilateral hemisphere compared with the sham group. HSPB8 overexpression markedly attenuated the EB leakage compared with ICH+ pLV-GFP group (p < 0.05). We observed the ultrastructure of BBB in the perihematomal brain tissues by TEM. In normal condition, basal laminas were found to be continuous and integrate; the endothelial tight junctions (TJs) appeared as electron-dense linear structures. At 24h after ICH, the TJs of endothelial cells were seriously collapsed, which appeared as reduced electron density and endothelium detachment. HSPB8 overexpression notably reversed these damages (Figure 4B).

### HSPB8 increased the TJPs expression after ICH

**Figure 5:**
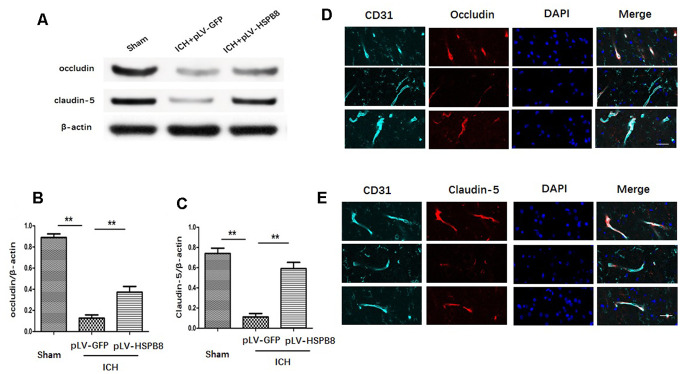
**HSPB8 overexpression reduced neutrophil infiltration and proinflammatory cytokine production at 24h following ICH.** (**A**) Representative pictures of infiltrated neutrophils (MPO + cells) in peri-hematoma area of ipsilateral cortex 24h after perfusion following ICH. Nuclei were stained with DAPI. Scale bar = 50μm (n=5, mean ± SEM). (**B**, **C**). IL-1β and TNF-α secretions in perihematomal area were analyzed by ELISA (n=5, mean ± SEM). (**D**, **E**). mRNA of IL-1β and TNF-α in perihematomal area were analyzed by RT-PCR (n=5, mean ± SEM). **P <0.01 versus indicated group. n=number of animals per group.

The expression of occludin and claudin-5 was assessed by western blot and immunofluorescence staining. We observed that the level of occludin and claudin-5 was significantly decreased at 24h after ICH in comparison with the sham group. HSPB8 rescued the TJPs loss in perihematomal area (Figure 5A–5C). Consistently, immunofluorescence staining revealed that ICH induced occludin and claudin-5 junctional discontinuity compared with the control group. HSPB8 overexpression attenuated ICH-induced degradation of occludin and claudin-5 (Figure 5D–5E).

### HSPB8 overexpression alleviated hemorrhagic inflammatory injury 24h post-ICH

**Figure 6:**
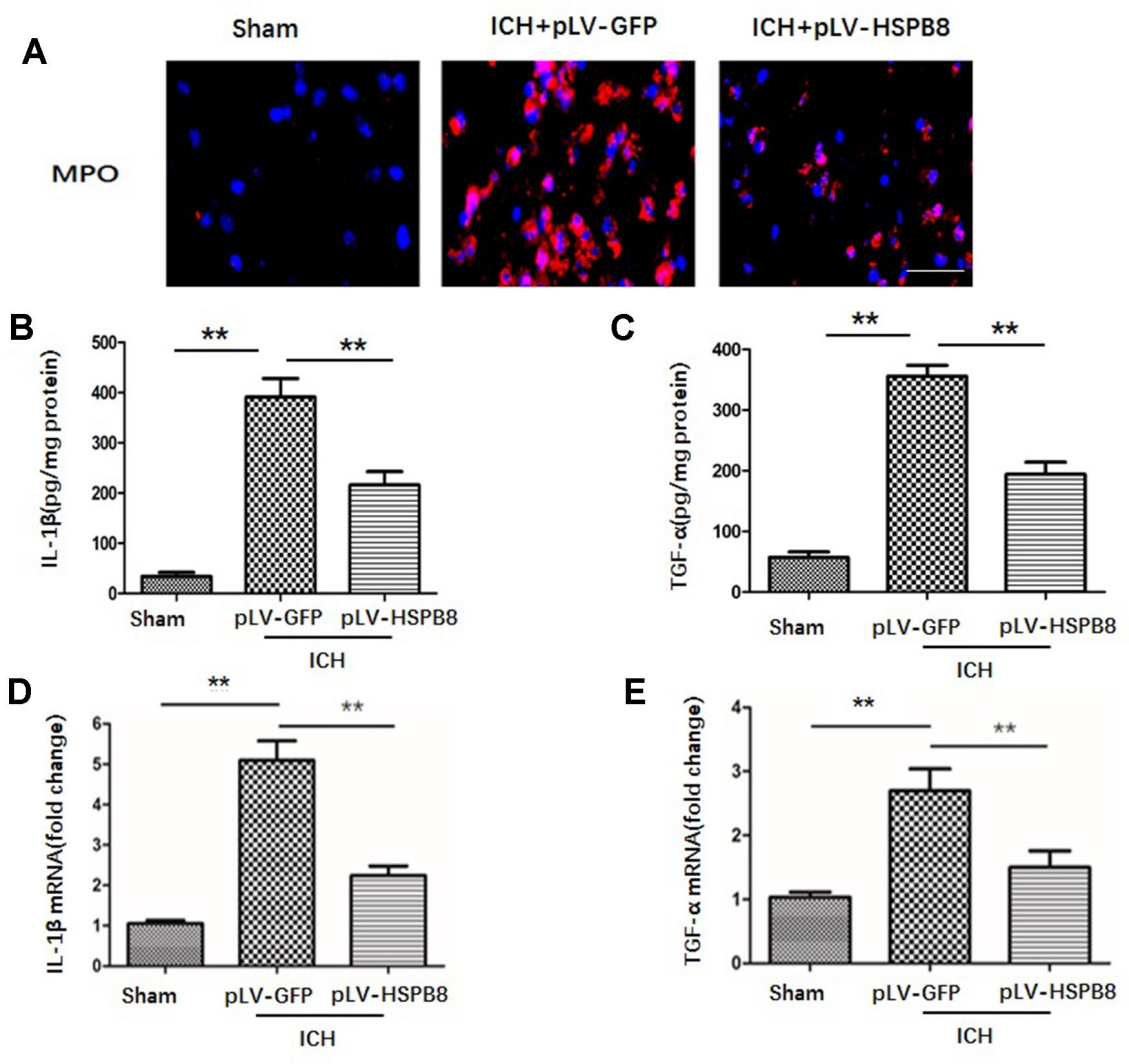
**HSPB8 overexpression protected against ICH-induced TJPs degradation.** (**A**) Occludin and claudin-5 expressions were measured by western blot. (**B**, **C**) Quantification of occludin and claudin-5 (n=5, mean ± SEM). (**D**, **E**) Representative images of occludin and claudin-5 positive vessels. Scale bar=50 μm (n=5, mean± SEM). **P <0.01 versus indicated group. n=number of animals per group.

BBB compromise has been regarded as a key event in ICH-induced SBI through an orchestrated series of events to facilitate passage of the leukocyte into brain tissue, which subsequently exacerbates neuroinflammatory response [17]. Those inflammatory processes determine the severity and prognosis of ICH. Therefore, we then examined the effect of HSPB8 overexpression on the leukocyte infiltration at 24h after ICH. As depicted in Figure 6A, immunofluorescence staining of 24h after ICH revealed that significant presence of MPO+ neutrophils in the perihematomal tissue, which markedly attenuated by HSPB8 overexpression. To evaluate the neuroinflammatory response, pro-inflammatory cytokines, including tumor necrosis factor-α (TNF-α) and interleukin-1β (IL-1β), in perihematomal tissue were quantified by ELISA. As indicated in Figure 6B, 6C, HSPB8 suppressed ICH-induced proinflammatory cytokines secretion. Consistently, relative mRNA transcript levels of inflammatory cytokines in perihematomal regions increased markedly 24h after ICH, compared with sham group. HSPB8 overexpression attenuated the elevated mRNA level of TNF-α and IL-1β after ICH (Figure 6D, 6E). These results indicated that HSPB8 suppressed the inflammatory responses following ICH.

### HSPB8 activated the Akt/GSK3β/β-catenin pathway following ICH

**Figure 7:**
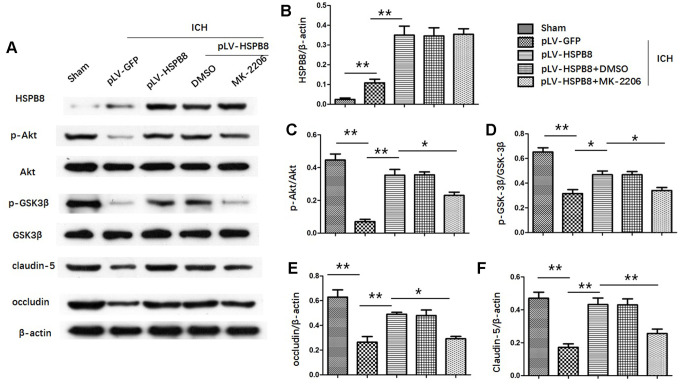
**HSPB8 activated Akt/GSK3β pathway and Akt inhibition abolished the restorative effect of HSPB8 on TJPs.** (**A**) Western blot analysis was performed to measure the protein levels of HSPB8, p-Akt, Akt, p-GSK3β, GSK3β, occludin and claudin-5. (**B**–**F**) Quantitative analysis of western blot analyses (n=6, mean± SEM). *P <0.05 versus indicated group; **P <0.01 versus indicated group. n=number of animals per group.

To determine Akt activation, the phosphorylation of Akt at S473 was measured. In the *in vivo* ICH model, the level of total Akt did not change, whereas the p-Akt (S473) in the perihematomal area was decreased at 24h after ICH compared with sham group. HSPB8 overexpression reversed ICH-induced reduction of p-Akt (Figure 7A, 7C).

**Figure 8:**
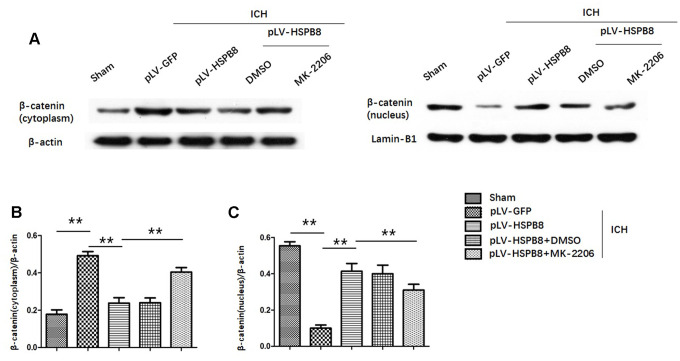
**HSPB8 promoted β-catenin nuclear translocation and MK2206 reversed this effect.** (**A**) Representative western blot bands of β-catenin in cytoplasm (left panel) and nucleus (right panel). (**B**, **C**) Quantification of plasm-protein HSPB8 and nucleoprotein HSPB8 (n=6, mean± SEM). **P <0.01 versus indicated group. n=number of animals per group.

Likewise, levels of p-GSK3β(ser9) were significantly higher in HSPB8-treated group than in the vehicle-treated group at 24h after ICH (Figure 7A, 7D). Moreover, compared with sham group, plasm-protein β-catenin was increased and nucleoprotein β-catenin was decreased in the ICH+ pLV-GFP group, while HSPB8 overexpression significantly increased nuclear accumulation of β-catenin and led to a corresponding decrease of β-catenin in the cytoplasm (Figure 8A–8D).

### Neuroprotective effect of HSPB8 acted through activating Akt/GSK3β/β-catenin pathway

**Figure 9:**
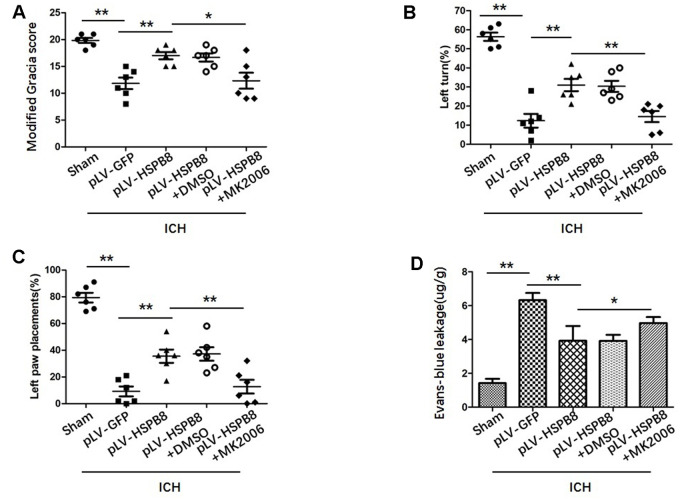
**MK2206 attenuated the protective effects of HSPB8.** (**A**–**C**) Modified Garcia test, corner turn test, and limb placement test in sham, ICH+pLV-GFP, ICH+pLV-HSPB8, ICH+pLV-HSPB8+DMSO, ICH+pLV-HSPB8 + MK2206 groups (n=6, mean±SEM). (**D**) Evans blue dye extravasation in different groups (n=6, mean±SEM) *P <0.05 versus indicated group; **P <0.01 versus indicated group. n=number of animals per group.

To explore whether HSPB8 exerted its neuroprotective effects via modulating Akt/GSK3β/β-catenin pathway, Akt-specific inhibitor MK2206 was administrated at 1h after ICH induction. Western blot results showed that HSPB8 overexpression was significantly upregulated at 24h after ICH. Administration of MK2206 did not obviously affect the HSPB8 expression (Figure 7A, 7B). Western blotting analysis documented downregulation of Akt phosphorylation after MK2206 treatment, as well as reduction of its downstream targets, p-GSK3β, and nuclear β-catenin after ICH (P < 0.05, ICH +pLV-GFP+DMSO vs ICH +pLV-GFP+MK2206, Figure 7A, 7C, 7D, Figure 8A–8D). At 24h post-ICH, HSPB8 overexpression significantly improved neurological function and reduced the EB leakage, and this neuroprotective effect was greatly abolished by MK2206 (Figure 9A–9C). Additionally, HSPB8 prevented ICH-induced reduction of TJPs, and MK2206 abrogated this effect of HSPB8 (Figure 6A, Figure 9D).

## DISCUSSION

The aim of the first experiment was to examine the time course and spatial expression in perihematomal area after ICH. In our study, HSPB8 expression was significantly upregulated and peaked at 24h after ICH. Double immunofluorescence staining revealed that HSPB8 immunoreactivity was found sparsely in peri-hematoma GFAP-positive astrocytes and CD31-positive endothelial cells. ICH significantly increased expression of HSPB8 in these BBB elements. HSPB8 co-localized with endothelial cells and astrocytes and was upregulated after ICH, indicating that HSPB8 may play a significant role in regulating BBB permeability after ICH.

To investigate the role of HSPB8 after the induction of ICH, we successfully overexpressed HSPB8 in rat local brain tissues with lentivirus system through *i.c.v* injection. pLV-HSPB8 administration significantly improved neurobehavioral functions and reduced brain edema after ICH.

Composed of highly specialized brain endothelial cells (ECs), BBB maintains the hemostasis of neuronal microenvironment [15]. BBB disruption is a crucial initiating contributor to ICH-induced SBI [16]. In the present study, therefore, the mechanism underlying the effect of HSPB8 after ICH was focused on BBB protection. Lentivirus *i.c.v* injection induced HSPB8 overexpression attenuated EB extravasation induced by ICH. In accordance with this result, TEM revealed that HSPB8 overexpression reversed the discontinuity of TJs to some extent. TJs consist of several transmembrane proteins and membrane-associated cytoplasmic proteins [17, 18]. Mounting evidence support that loss of TJPs results in BBB hyperpermeability and subsequent SBI after ICH [19]. In our study, HSPB8 overexpression obviously protected against reduction and disorganization of TJPs, including claudin-5 and occludin. TJ disorganization leads to leukocyte infiltration and thus triggers inflammation [20]. Inflammation cascade ultimately promotes neuronal cell death and leads to neurological deterioration. MPO, an indicator of neutrophil infiltration [21], was employed in the present study. In our study, HSPB8 overexpression mitigated neutrophil infiltration as measured by immunofluorescence, and downregulated the expression of TNF-α, IL-1β, measured by ELISA and western-blot at 24h after ICH. Thus, our study provided evidence that HSPB8 efficiently preserved BBB integrity and alleviated neuroinflammation after ICH.

Akt is a serine/threonine kinase and a primary downstream effector of phosphatidylinositol -3 kinase (PI3K). Akt coordinates a constellation of intracellular signals and is involved in multiple biological functions, including cell proliferation and survival. After phosphorylation, Akt phosphorylates glycogen synthase kinase-3β(GSK3β) at the S9 residue, which suppresses GSK3β activity [22]. Inactivated GSK-3β leads to β-catenin translocation to the nucleus [23], and thus protects against the disruption of BBB through controlling the expression of multiple downstream targets such as occludin and claudin-5 [24, 25]. In this study, we demonstrated that HSPB8 overexpression led to a significant increase in phosphorylation of Akt and GSK3β, as well as promoted cytoplasm-nucleus translocation of β-catenin at 24h following ICH. Therefore, activating Akt/GSK-3β/β-catenin signaling pathway may be involved in the positive role of HSPB8 after ICH. MK2206, a specific inhibitor of Akt, was then chosen to evaluate the precise mechanism underlying the HSPB8’s protective effect. MK2206 inhibited the expression of p-Akt, p-GSK-3β and nuclear translocation of β-catenin. Additionally, MK2206 significantly reversed the suppressive effect of HSPB8 on neuronal injury and BBB disruption, and the restorative effect of HSPB8 on claudin-5 and occludin at 24h after ICH. The results demonstrated that HSPB8 alleviated ICH-induced brain injury and BBB disruption through Akt/ GSK3β /β-catenin signaling pathway. However, there are some limitations in the current study. Akt signaling is modulated by a complex network of regulatory proteins during neurological disease processes, often involving crosstalk with scenarios of molecular pathways. Therefore, further studies need to be conducted to elucidate the precise mechanism of HSPB8 in Akt/GSK3β/β-catenin signaling modulation after ICH.

In conclusion, our study demonstrated that HSPB8 overexpression ameliorated brain damage in the early injury stages after ICH via maintaining BBB integrity. Activation of Akt/GSK3β/β-catenin signaling pathway underlies the protective effects of HSPB8. Therefore, HSPB8-based therapies hold a great promise against ICH progression.

## MATERIALS AND METHODS

### Experimental design

**Figure 10:**
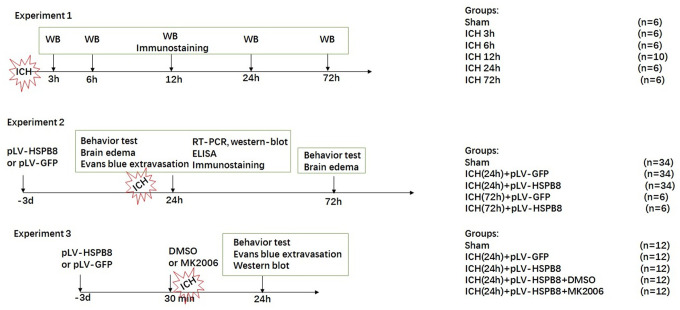
**Experimental design and animal groups.**

The study was divided into three parts as sketched in Figure 10. In experiment 1, 36 rats were randomly divided into six groups (n=6 for each group): sham, 3h, 6h, 12h, 24h and 72h after ICH, for western blot. 8 additional rats in the sham and 24 h groups(n=4) were used for double immunofluorescence staining of HSPB8 combined with specific markers for astrocytes, and microvessels.

In experiment 2, 114 rats randomly divided into five groups: 1) sham (n = 34), 2) ICH (24h) + pLV-GFP (n = 34), 3) ICH(24h) + pLV-HSPB8(n = 34), 4) ICH (72h) + pLV-GFP (n = 6), 5) ICH(72h) + pLV-HSPB8(n = 6). pLV-GFP or pLV-HSPB8 was administered intracerebroventricularly 72h before ICH. Neurobehavior and brain edema (*n*=6), Evans-blue leakage (*n* = 8), enzyme-linked immunosorbent assay (ELISA) (*n* = 5), western-blot(*n* = 5), real-time reverse transcription-polymerase chain reaction (RT-PCR) (*n* = 5) and immunofluorescence staining of MPO, claudin-5 and occludin (*n* = 5) were applied to explore the role of HSPB8 in ICH

In experiment 3, 60 rats were distributed randomly into five groups:1) sham (*n* = 12), 2) ICH + pLV-GFP (*n* = 12), 3) ICH + pLV-HSPB8 (*n* = 12), 4) ICH + pLV-HSPB8+ DMSO (*n* = 12), 5) ICH + pLV-HSPB8+ MK2206 (*n* = 12). 5 μl of the DMSO and MK2206 were infused were applied intracerebroventricularly 30 min before ICH induction in ICH + pLV-HSPB8+ DMSO, ICH + pLV-HSPB8+ MK2206 rats, respectively. Neurobehavior and evans-blue analysis (n = 6), western-blot (n = 6) were used to explore the underlying mechanisms. All animals in our study were decapitated for sample collection under deep anesthesia.

### Animals

Male Sprague–Dawley (SD) rats (4-6 weeks old) purchased from Shanghai Laboratory Animal Center were used in the experiment. All experimental procedures were authorized by the Institutional Animal Care and Use Committee of The Second Xiangya Hospital, Central South University. The ethical approval reference number is 2017-204. The rats were acclimated in the animal center and fed *ad libitum*. All procedures were carried out during day time (light cycle for animals).

### Intracerebroventricular (i.c.v) injection

Three days before ICH induction, rats were anesthetized, and then i.c.v injection was performed as previously described [26]. Briefly,1 hole was drilled (Stereotaxic coordinates:1.5 mm anteroposterior to the bregma, 1.8 mm mediolateral to the midline). pLV-HSPB8 or pLV-GFP (control) was then delivered into the right lateral cerebral ventricle through a hypodermic needle connected to a Hamilton microliter syringe (5μl, at the rate of 0.5 μl/min). The needle was remained in place for an additional 5 min after injection and then removed slowly.

### ICH model

Animal model of ICH was conducted based on a previous study. After anesthesia with 10% chloral hydrate (3 ml/kg, intraperitoneal injection) was used to anesthetize the rats. Under deep anesthesia, the animals were fixed on a rat brain stereotactic apparatus. Stereotaxic injection of collagenase type VII-S (0.3 U in 2 μl sterile normal saline) was infused into right striatum at the following coordinates: 0.2 mm anterior, 3.5 mm right lateral, and 6 mm ventral to the bregma. The needle was withdrawn after 10 minutes. The sham-operated animals received an operation with intracerebrally injection with equal volume of sterile saline.

### Neurological deficit scoring evaluation

Neurological functions of the animals were examined and scored blindly at 24h and 72h after ICH. Garcia test consisted of seven parameters: spontaneous activity, symmetry of limb movement, vibrissae touch, axial sensation, lateral turning, forelimb outstretch, and climbing. In the corner turn test, rats approached into a 30° corner. In order to exit, they had to turn around the corner, either turning to the left or right. The choice of left turns for a total of 10 trials was recorded. In the forelimb placing test, the contralesional vibrissae of the rat were swept, and the number of quick placement of the ipsilateral forelimb to the vibrissa stimulation was recorded. The test was repeated 10 times and the percent of ipsilateral forelimb placement was calculated.

### Measurement of brain water content

Brain water content was measured via a wet/dry weight method, according to previous literature. Briefly, at 24h and 72h after ICH, the brains of rats were removed immediately after anesthetization and separated into five parts: ipsilateral and contralateral cortex, ipsilateral and contralateral basal ganglia, and cerebellum. Each part was weighed immediately to obtain wet weight (ww). After being dried at 100 °C for 72 h, dry weight (DW) of brain slices was obtained. Brain water content (BWC) was calculated as: [(WW−DW)/(WW)] × 100%.

### Evans blue extravasation

Evans blue (EB) extravasation was used to measure the integrity BBB permeability as previously described [27]. Briefly, 2% evans blue dye (4 ml/kg) was injected via intraperitoneal route. After 2 hour circulation, rats were transcardially perfused with 0.1 M cold PBS. Afterwards, brain samples were weighed, homogenized in PBS, sonicated and then centrifuged (15,000*g*, 30 min). 0.5 mL of the supernatant was then homogenized in an equal volume of 60% trichloroacetic acid. The absorbency of the supernatant at 620 nm was measured using a spectrophotometer. The tissue content of EB was quantified according to a standard curve.

### Western blotting

Nuclear and cytoplasmic fractions were prepared using NE-PER nuclear and cytoplasmic extraction reagents following manufacturer’s protocol. Equal amounts of extracted proteins were separated using 10% SDS-PAGE and then electrotransferred to PVDF membranes. The membranes were blocked in TBST containing 5% non-fat milk for 2 hours and blotted overnight with antibodies against occludin (1 : 1000, Cat# ab216327, Abcam), claudin-5 (1:1000, Cat# ab131259, Abcam), HSPB8(1:1000, Cat# MA5-32421, Thermo fisher Scientific), p-Akt(1:1000, Cat# ab81283, Abcam), Akt (1: 1000, Cat# ab179463, Abcam), p-GSK-3β (1:1000, Cat# ab75814, Abcam), GSK-3β (1:1000, Cat#ab93926, Abcam Cambridge), and β-actin(1:1000, Cat# 12262, CST). Afterwards, the membranes were washed and incubated for 1 h with secondary antibodies in the room temperature. Immunoblots were visualized using an enhanced chemiluminescence (ECL) system (Thermo Scientific).

### Immunofluorescence staining

Immunofluorescence studies were performed as previously described [28]. After being anesthetized with chloral hydrate after 24h post-ICH, rats were transcardially perfused with ice-cold PBS followed by 10% paraformaldehyde. The brains were collected and post-fixed in 4% paraformaldehyde for another 24h, and then cryoprotected in 25% sucrose until tissue sink. Afterwards, 20-μm- thick brain coronal sections were obtained using a cryostat. Brain sections were then incubated with following primary antibodies at 4°C overnight: anti-HSPB8(1:100, Thermo Fisher Scientific), anti-occludin (1:100, Abcam), anti-claudin-5 (1:100, Abcam), anti-MPO (1:100, Cat# ab9535, Abcam), anti-NeuN (1:100, Cat# ab177487, Abcam). After thorough washes in PBS, the specimens were incubated with Alexa 488 secondary antibodies (1:1000, Cat# A32723, Thermo Fisher Scientific) or Alexa 594 secondary antibodies (1:500, Cat # A-11032, Thermo Fisher Scientific). Nuclei were stained with DAPI (1:500, Cat# D3571, Thermo Fisher Scientific).

### ELISA

The rats were killed 24h after ICH, expression of inflammation markers, including TNF-α and IL-1β, from perihematomal tissue extracts was measured by respective ELISA kits (R&D Systems), according to manufacturer’s instructions.

### Real-time PCR (RT-PCR)

Total RNA from perihematomal brain tissues was extracted using Trizol reagent (Invitrogen, Life Technologies). Total RNA was reverse transcribed to generate first-strand cDNA using a PrimeScript RT reagent kit (Invitrogen). The real-time PCR primers used in this study are listed in Table 1. Afterwards, RT-PCR was performed in a 10 μl final reaction volume using the miScriptSYBR®green PCR Kit (Qiagen, USA). The mRNA level of each target gene was normalized to GADPH mRNA. Relative levels of gene expression were calculated using 2−ΔΔCt.

**Table 1:** Primer sequences for target genes.

**Gene**	**Forward (5′-3′)**	**Reverse (5′-3′)**
IL-1β	GCAACTGTTCCTGAACTCAACT	ATCTTTTGGGGCGTCAACT
TNF-α	CCCTCACACTCAGATCATCTTCT	GCTACGACGTGGGCTACAG
GADPH	CAATGTGTCCGTCGTGGATCT	GTCCTCAGTGTAGCCCAAGATG

### Statistical analysis

Data were expressed as the means ± SEM. All analyses were performed using Prism software (GraphPad, CA, USA). Differences between groups were analyzed by either Student’s t test or one-way analysis of variance (ANOVA) and Tukey's posthoc test. A value of P < 0.05 was considered statistically significant.

## References

[1] Steiner T, Al-Shahi Salman R, Beer R, Christensen H, Cordonnier C, Csiba L, Forsting M, Harnof S, Klijn CJ, Krieger D, Mendelow AD, Molina C, Montaner J, et al, and European Stroke Organisation. European Stroke Organisation (ESO) guidelines for the management of spontaneous intracerebral hemorrhage. Int J Stroke. 2014; 9:840–55. 10.1111/ijs.12309 25156220

[2] Krishnamurthi RV, Moran AE, Forouzanfar MH, Bennett DA, Mensah GA, Lawes CM, Barker-Collo S, Connor M, Roth GA, Sacco R, Ezzati M, Naghavi M, Murray CJ, Feigin VL, Global Burden of Diseases, Injuries, and Risk Factors 2010 Study Stroke Expert Group. The global burden of hemorrhagic stroke: a summary of findings from the GBD 2010 study. Glob Heart. 2014; 9:101–06. 10.1016/j.gheart.2014.01.003 25432119

[3] Crippa V, Carra S, Rusmini P, Sau D, Bolzoni E, Bendotti C, De Biasi S, Poletti A. A role of small heat shock protein B8 (HspB8) in the autophagic removal of misfolded proteins responsible for neurodegenerative diseases. Autophagy. 2010; 6:958–60. 10.4161/auto.6.7.13042 20699640

[4] Crippa V, Sau D, Rusmini P, Boncoraglio A, Onesto E, Bolzoni E, Galbiati M, Fontana E, Marino M, Carra S, Bendotti C, De Biasi S, Poletti A. The small heat shock protein B8 (HspB8) promotes autophagic removal of misfolded proteins involved in amyotrophic lateral sclerosis (ALS). Hum Mol Genet. 2010; 19:3440–56. 10.1093/hmg/ddq257 20570967

[5] Li F, Yang B, Li T, Gong X, Zhou F, Hu Z. HSPB8 over-expression prevents disruption of blood-brain barrier by promoting autophagic flux after cerebral ischemia/reperfusion injury. J Neurochem. 2019; 148:97–113. 10.1111/jnc.14626 30422312

[6] Paolinelli R, Corada M, Orsenigo F, Dejana E. The molecular basis of the blood brain barrier differentiation and maintenance. Is it still a mystery? Pharmacol Res. 2011; 63:165–71. 10.1016/j.phrs.2010.11.012 21167284

[7] Tran KA, Zhang X, Predescu D, Huang X, Machado RF, Göthert JR, Malik AB, Valyi-Nagy T, Zhao YY. Endothelial β-catenin signaling is required for maintaining adult blood-brain barrier integrity and central nervous system homeostasis. Circulation. 2016; 133:177–86. 10.1161/CIRCULATIONAHA.115.015982 26538583 PMC4814374

[8] Clevers H, Nusse R. Wnt/β-catenin signaling and disease. Cell. 2012; 149:1192–205. 10.1016/j.cell.2012.05.012 22682243

[9] Stamos JL, Weis WI. The β-catenin destruction complex. Cold Spring Harb Perspect Biol. 2013; 5:a007898. 10.1101/cshperspect.a007898 23169527 PMC3579403

[10] Cross DA, Alessi DR, Cohen P, Andjelkovich M, Hemmings BA. Inhibition of glycogen synthase kinase-3 by insulin mediated by protein kinase B. Nature. 1995; 378:785–89. 10.1038/378785a0 8524413

[11] Hu Z, Yang B, Mo X, Zhou F. HspB8 mediates neuroprotection against OGD/R in N2A cells through the phosphoinositide 3-kinase/Akt pathway. Brain Res. 2016; 1644:15–21. 10.1016/j.brainres.2016.05.012 27178361

[12] Lizano P, Rashed E, Kang H, Dai H, Sui X, Yan L, Qiu H, Depre C. The valosin-containing protein promotes cardiac survival through the inducible isoform of nitric oxide synthase. Cardiovasc Res. 2013; 99:685–93. 10.1093/cvr/cvt136 23737493 PMC3746953

[13] Matsushima-Nishiwaki R, Toyoda H, Takamatsu R, Yasuda E, Okuda S, Maeda A, Kaneoka Y, Yoshimi N, Kumada T, Kozawa O. Heat shock protein 22 (HSPB8) reduces the migration of hepatocellular carcinoma cells through the suppression of the phosphoinositide 3-kinase (PI3K)/AKT pathway. Biochim Biophys Acta Mol Basis Dis. 2017; 1863:1629–39. 10.1016/j.bbadis.2017.04.021 28456666

[14] Kuroiwa T, Cahn R, Juhler M, Goping G, Campbell G, Klatzo I. Role of extracellular proteins in the dynamics of vasogenic brain edema. Acta Neuropathol. 1985; 66:3–11. 10.1007/BF00698288 3993334

[15] Zhao Z, Nelson AR, Betsholtz C, Zlokovic BV. Establishment and dysfunction of the blood-brain barrier. Cell. 2015; 163:1064–78. 10.1016/j.cell.2015.10.067 26590417 PMC4655822

[16] Xi G, Keep RF, Hoff JT. Mechanisms of brain injury after intracerebral haemorrhage. Lancet Neurol. 2006; 5:53–63. 10.1016/S1474-4422(05)70283-0 16361023

[17] Hawkins BT, Davis TP. The blood-brain barrier/neurovascular unit in health and disease. Pharmacol Rev. 2005; 57:173–85. 10.1124/pr.57.2.4 15914466

[18] Piontek J, Winkler L, Wolburg H, Müller SL, Zuleger N, Piehl C, Wiesner B, Krause G, Blasig IE. Formation of tight junction: determinants of homophilic interaction between classic claudins. FASEB J. 2008; 22:146–58. 10.1096/fj.07-8319com 17761522

[19] Jiao H, Wang Z, Liu Y, Wang P, Xue Y. Specific role of tight junction proteins claudin-5, occludin, and ZO-1 of the blood-brain barrier in a focal cerebral ischemic insult. J Mol Neurosci. 2011; 44:130–39. 10.1007/s12031-011-9496-4 21318404

[20] Shastri A, Bonifati DM, Kishore U. Innate immunity and neuroinflammation. Mediators Inflamm. 2013; 2013:342931. 10.1155/2013/342931 23843682 PMC3697414

[21] Gong C, Hoff JT, Keep RF. Acute inflammatory reaction following experimental intracerebral hemorrhage in rat. Brain Res. 2000; 871:57–65. 10.1016/s0006-8993(00)02427-6 10882783

[22] Kaidanovich-Beilin O, Woodgett JR. GSK-3: functional insights from cell biology and animal models. Front Mol Neurosci. 2011; 4:40. 10.3389/fnmol.2011.00040 22110425 PMC3217193

[23] Sato N, Meijer L, Skaltsounis L, Greengard P, Brivanlou AH. Maintenance of pluripotency in human and mouse embryonic stem cells through activation of Wnt signaling by a pharmacological GSK-3-specific inhibitor. Nat Med. 2004; 10:55–63. 10.1038/nm979 14702635

[24] Liebner S, Dijkhuizen RM, Reiss Y, Plate KH, Agalliu D, Constantin G. Functional morphology of the blood-brain barrier in health and disease. Acta Neuropathol. 2018; 135:311–36. 10.1007/s00401-018-1815-1 29411111 PMC6781630

[25] Ramirez SH, Fan S, Dykstra H, Rom S, Mercer A, Reichenbach NL, Gofman L, Persidsky Y. Inhibition of glycogen synthase kinase 3β promotes tight junction stability in brain endothelial cells by half-life extension of occludin and claudin-5. PLoS One. 2013; 8:e55972. 10.1371/journal.pone.0055972 23418486 PMC3572160

[26] Yeo JF, Ong WY, Ling SF, Farooqui AA. Intracerebroventricular injection of phospholipases A2 inhibitors modulates allodynia after facial carrageenan injection in mice. Pain. 2004; 112:148–55. 10.1016/j.pain.2004.08.009 15494195

[27] Belayev L, Busto R, Ikeda M, Rubin LL, Kajiwara A, Morgan L, Ginsberg MD. Protection against blood-brain barrier disruption in focal cerebral ischemia by the type IV phosphodiesterase inhibitor BBB022: a quantitative study. Brain Res. 1998; 787:277–85. 10.1016/s0006-8993(97)01499-6 9518648

[28] Xie Z, Huang L, Enkhjargal B, Reis C, Wan W, Tang J, Cheng Y, Zhang JH. Intranasal administration of recombinant netrin-1 attenuates neuronal apoptosis by activating DCC/APPL-1/AKT signaling pathway after subarachnoid hemorrhage in rats. Neuropharmacology. 2017; 119:123–33. 10.1016/j.neuropharm.2017.03.025 28347836 PMC5490977

